# Applications of Artificial Intelligence and Machine Learning in Spine MRI

**DOI:** 10.3390/bioengineering11090894

**Published:** 2024-09-05

**Authors:** Aric Lee, Wilson Ong, Andrew Makmur, Yong Han Ting, Wei Chuan Tan, Shi Wei Desmond Lim, Xi Zhen Low, Jonathan Jiong Hao Tan, Naresh Kumar, James T. P. D. Hallinan

**Affiliations:** 1 Department of Diagnostic Imaging, National University Hospital, 5 Lower Kent Ridge Rd, Singapore 119074, Singapore; wilson.ong@mohh.com.sg (W.O.); andrew_makmur@nuhs.edu.sg (A.M.); yonghan_ting@nuhs.edu.sg (Y.H.T.); weichuan.tan@mohh.com.sg (W.C.T.); desmond.lim@mohh.com.sg (S.W.D.L.); xi_zhen_low@nuhs.edu.sg (X.Z.L.); james_hallinan@nuhs.edu.sg (J.T.P.D.H.); 2 Department of Diagnostic Radiology, Yong Loo Lin School of Medicine, National University of Singapore, 10 Medical Drive, Singapore 117597, Singapore; 3 National University Spine Institute, Department of Orthopaedic Surgery, National University Health System, 1E Lower Kent Ridge Road, Singapore 119228, Singapore; jonathan_jh_tan@nuhs.edu.sg (J.J.H.T.); dosksn@nus.edu.sg (N.K.)

**Keywords:** artificial intelligence, machine learning, spine, spinal cord, magnetic resonance imaging

## Abstract

Diagnostic imaging, particularly MRI, plays a key role in the evaluation of many spine pathologies. Recent progress in artificial intelligence and its subset, machine learning, has led to many applications within spine MRI, which we sought to examine in this review. A literature search of the major databases (PubMed, MEDLINE, Web of Science, ClinicalTrials.gov) was conducted according to the Preferred Reporting Items for Systematic Reviews and Meta-Analyses (PRISMA) guidelines. The search yielded 1226 results, of which 50 studies were selected for inclusion. Key data from these studies were extracted. Studies were categorized thematically into the following: Image Acquisition and Processing, Segmentation, Diagnosis and Treatment Planning, and Patient Selection and Prognostication. Gaps in the literature and the proposed areas of future research are discussed. Current research demonstrates the ability of artificial intelligence to improve various aspects of this field, from image acquisition to analysis and clinical care. We also acknowledge the limitations of current technology. Future work will require collaborative efforts in order to fully exploit new technologies while addressing the practical challenges of generalizability and implementation. In particular, the use of foundation models and large-language models in spine MRI is a promising area, warranting further research. Studies assessing model performance in real-world clinical settings will also help uncover unintended consequences and maximize the benefits for patient care.

## 1. Introduction

The spine is an important site of pathology and can be affected by a variety of conditions, including degenerative, neoplastic, infectious, traumatic and inflammatory demyelinating diseases. Diagnostic imaging often plays a key role in the diagnosis of spinal diseases. In addition, imaging is also vital for planning treatments such as surgery and minimally invasive procedures as it allows for the localization and quantification of underlying pathologies [1].

Various imaging modalities can be used in spine imaging. Radiographs often provide initial assessment of symptoms that may be attributed to a spinal pathology, such as neck or back pain, radiculopathy, or myelopathy. They are a cost-efficient and widely available diagnostic tool that can provide rapid assessment of spinal alignment, fractures, and degenerative changes. Erosive changes can also be detected and may suggest the presence of neoplasms or underlying infection, albeit with a relatively low sensitivity. Radiographs also offer a relatively low-cost method for the dynamic assessment of spinal instability [1,2]. Computed tomography (CT) provides a superior delineation of complex spinal anatomy, which can be challenging to accurately assess using radiographs. In the setting of trauma, CT is the modality of choice for evaluating fractures and dislocations in the cervical spine as it allows for the rapid imaging of patients who may have significant traumatic injuries and be in an unstable clinical condition. It also allows for a good visualization of the cortical bone [3]. CT scans can also be used for pre-operative planning as certain pathologies, such as ossification of the posterior longitudinal ligament (OPLL), are readily visualized [4].

While radiographs and CT remain significant imaging tools, magnetic resonance imaging (MRI) has surpassed both in the range of pathologies that it is able to image. MRI scans have the advantage of being able to evaluate bone marrow signal thus allowing for an accurate detection of pathologies that alter the normal bone marrow, such as fractures or contusions, neoplastic disease or infection. In addition, MRI scans provide superior evaluation of soft tissue structures, such as the intervertebral disc, spinal ligaments, as well as the spinal cord and surrounding dural and epidural spaces [5,6,7]. Thus, MRIs have become widely recognized as the preferred modality to evaluate many spinal pathologies. CT myelography is an alternative modality used to assess the spinal cord and neural foramina. However, it requires the injection of contrast material into the spinal canal via lumbar puncture, making it more invasive and less widely used [8]. It is typically reserved for cases with MRI contraindications, such as patients with incompatible pacemakers.

Despite its many advantages, an important limitation of MRI is its relatively long acquisition times. To accommodate a growing number of scans, more time-efficient MRI pulse sequences have been developed. However, there is often a trade-off between diagnostic quality and time savings, resulting in faster sequences with lower resolution or tissue contrast [9]. More recent developments such as parallel imaging and compressed sensing have partially mitigated this [10,11], but scan time remains a pertinent issue. In addition, interpreting these MRIs can be a tedious and time-consuming process for the reporting radiologist. Each spinal level must be carefully examined for evidence of pathology. Additionally, there is significant interobserver variability in evaluating the severity of observed pathology [12]. The lack of standardized grading systems, particularly in the cervical and thoracic spine, further complicates this process. Finally, certain spinal pathologies present diagnostic challenges due to overlapping imaging characteristics. Differentiating between various types of spinal neoplasms or infections can be particularly difficult, especially for inexperienced radiologists, potentially impacting subsequent treatment decisions.

Artificial intelligence (AI) has been increasingly explored as a solution to many of these challenges, with widespread applications across medicine. Machine learning (ML) is a subset of AI that utilizes a combination of algorithms and statistical models to make predictions on new data [13,14,15]. Deep learning (DL) is a further subset of ML which has garnered significant interest in recent years. Compared to other types of ML, DL algorithms are generally more complex, requiring larger amounts of data and computational power. Such algorithms have been developed with the promise of impacting various areas of radiology. Most algorithms in radiology are ‘supervised’ via labeled datasets. Using labels provided by human readers, the DL model learns to identify patterns in a dataset, and its performance is studied using a separate test/validation dataset [14,15]. The number of applications for AI in radiology, including its subset ML, has increased significantly over time, now spanning areas such as image interpretation, protocolling scans, and optimizing workflows. Additionally, many of the challenges in spine imaging are not unique. Various AI techniques have been successfully applied across a broad spectrum of tasks in radiology and medicine, including endoscopic image analysis and image feature fusion and enhancement [16,17,18]. Many of these advancements have helped inform the innovations in spine imaging AI.

While several studies have examined the use of AI in specific aspects of spine imaging, our goal is to provide a comprehensive overview of the full spectrum of use cases in spine MRI by examining the available literature on a wide variety of applications. Additionally, we aim to identify gaps in the existing literature and propose areas for future research in the field.

## 2. Materials and Methods

Given the anticipated large number of studies that would be extracted, a scoping review was performed to adequately represent the breadth and depth of the current literature.

### 2.1. Literature Search Strategy

We performed a literature search of the major databases (PubMed, MEDLINE, Web of Science, ClinicalTrials.gov) on 8 February 2024, according to the Preferred Reporting Items for Systematic Reviews and Meta-Analyses (PRISMA) guidelines. The following medical subject headings (MeSH) and keywords were utilized: (“artificial intelligence” OR “AI” OR “machine learning” OR “ML” OR “deep learning” OR “DL”) AND (“spine” OR “spinal cord” OR “cord” OR “vertebra” OR “vertebral column” OR “spinal column” OR “intervertebral disc” OR “intervertebral disk) AND (“MRI” OR “MR” OR “magnetic resonance imaging”). Limits were applied to include only English language studies from the past eight years.

### 2.2. Study Screening and Selection Criteria

A two-stage screening process was used. Studies were first screened independently by two authors (A.L. and W.O.) by title and abstract. A full text review was then performed for any potentially eligible studies. Any controversies at either stage were reviewed by a third author (J.T.P.D.H.).

The inclusion criteria were as follows: studies on the use of AI or ML on MRI images focusing on spine-related applications, English studies, and studies performed on human subjects. The exclusion criteria were as follows: non-original research (for example, review articles, editorial correspondence), unpublished work, conference abstracts, and case reports. Studies that primarily focused on other imaging modalities (for example, radiographs, CT, or nuclear medicine imaging) or other body regions were excluded.

### 2.3. Data Extraction and Reporting

The selected studies were extracted and compiled onto a spreadsheet using Microsoft Excel Version 16.81 (Microsoft Corporation, Washington DC, USA). The following data was extracted:

1. Study details: authorship, year of publication and journal name;
2. Application and primary outcome measure;
3. Study details: sample size, spine region studied, MRI sequences used;
4. Artificial intelligence technique used;
5. Key results and conclusion.

## 3. Results

### 3.1. Search Results

**Figure 1:**
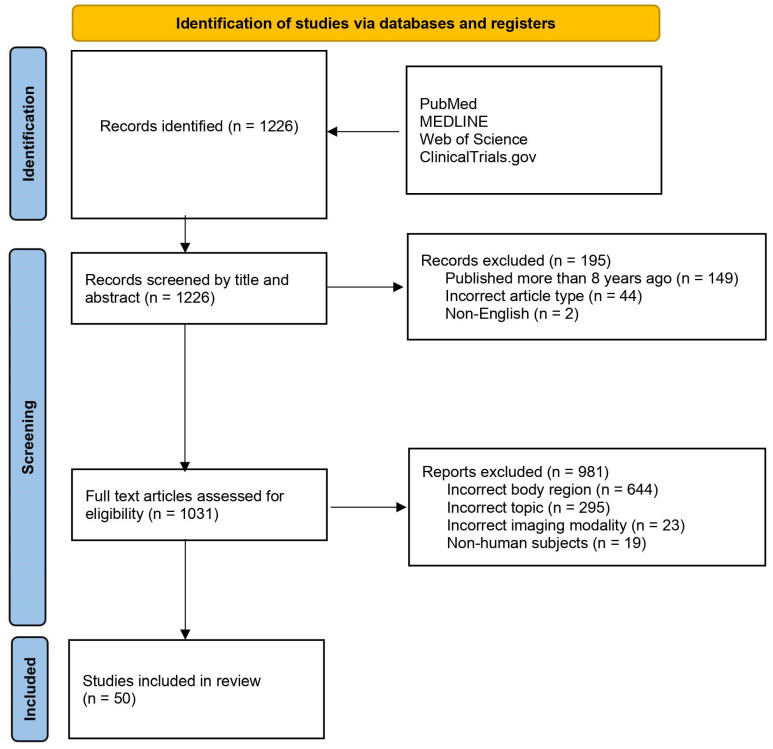
PRISMA flowchart showing the two-step study screening process. Adapted from PRISMA Group, 2020.

Our initial literature search identified 1226 studies, which were screened according to the specified criteria. Subsequently, 149 studies which did not meet the date range, 44 with an incorrect article type and 2 non-English language studies were initially excluded. This led to 1031 studies selected for full text screening, and the inclusion of 50 studies in the present review (see Figure 1 for a detailed flowchart). The studies are summarized in Table 1. Given the heterogeneity of the included studies, a formal meta-analysis could not be meaningfully performed.

**Figure 2:**
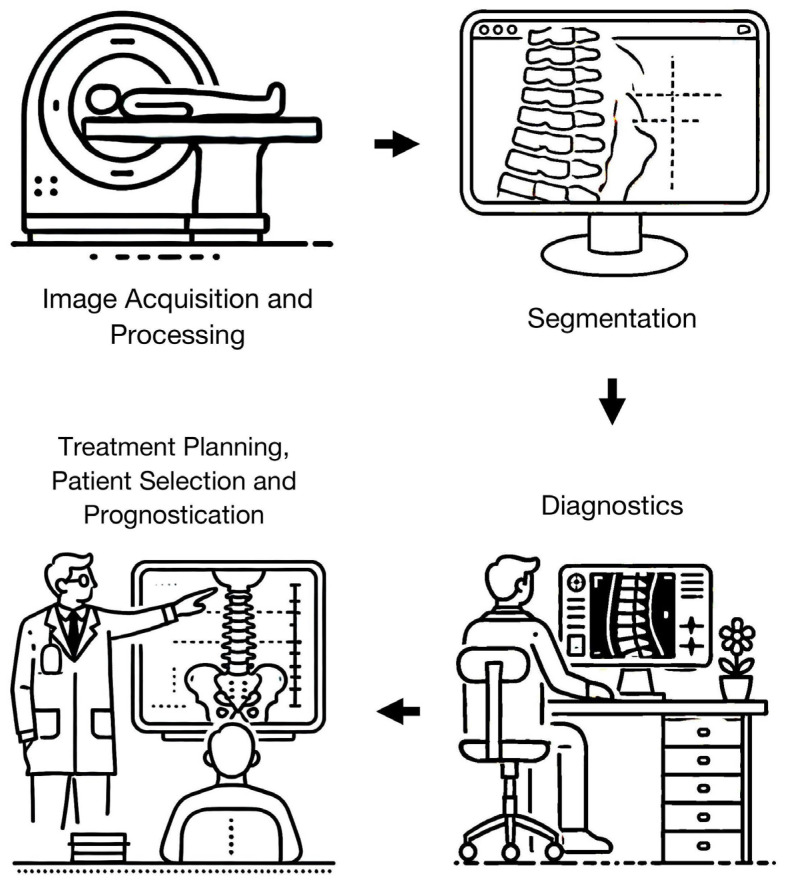
Key themes identified through the literature search representing potential areas where advances in artificial intelligence and machine learning can improve the field of spine MRI.

We classified the included studies based on the following themes: Image Acquisition and Processing (6/50, 12%), Segmentation (8/50, 16%), Diagnosis (27/50, 54%), Treatment Planning, Patient Selection and Prognosis (6/50, 12%) and Others (3, 6%) (Figure 2). We further sub-divided the Diagnosis theme into Degenerative (13/50, 26%), Neoplastic Diseases (7/50, 14%), Infection (3/50, 6%), Trauma (2/50, 4%), and Spondyloarthropathy (2/50, 4%).

### 3.2. Image Acquisition and Processing

AI has demonstrated promise in the area of image acquisition and processing, with multiple studies demonstrating the ability of deep learning (DL)-assisted acquisition and reconstruction techniques to reduce image acquisition times while maintaining similar image quality to conventional protocols. Kashiwagi et al. (2022) studied an ultrafast cervical spine MRI protocol (sagittal T1-, T2-weighted, short-tau inversion recovery (STIR), and axial T2*-weighted sequences) using a convolutional neural network (CNN)-based reconstruction which reduces the matrix size, oversampling rate, and number of excitations by applying a noise reduction algorithm. Scan quality was rated by three neuroradiologists, who graded various degenerative changes including central canal stenosis, foraminal stenosis and disc degeneration. Compared with a conventional MRI protocol, the DL-based reconstruction technique reduced scan time from 12 min 54 s to 2 min 57 s (achieving a time reduction of 9 min 57 s, 77% faster), with high levels of agreement (κ = 0.60–0.98) between the protocols [50]. In another study by Awan et al. (2024), the authors evaluated a lumbar spine MRI DL-accelerated protocol (sagittal T1-, T2-weighted, STIR, axial T2-weighted sequences) that was 57% faster compared to a conventional protocol (287 s versus 654 s). This protocol employs an unrolled variational network and neural networks to reduce the number of signal averages needed while preserving high image fidelity. The DL-accelerated protocol demonstrated non-inferiority for the assessment of foraminal and spinal canal stenosis, nerve compression, and facet arthropathy. However, there was increased artifact perception in the DL group. The authors proposed that further work could focus on other pathologies, such as spinal cord evaluation, to ensure the broad applicability of such protocols across various clinical scenarios [22]. Such protocols promise to generate significant time- and cost-savings for radiology departments. In addition, reducing examination time would potentially improve patient comfort, especially for those who may not be able to fully cooperate with long examination times due to pre-existing medical conditions or claustrophobia.

AI can also be applied to generate synthetic MRI sequences. In a multi-center trial, Tanenbaum et al. (2023) used existing sagittal T1- and T2-weighted MRI images to generate STIR images, which is the preferred MRI sequence to assess certain pathologies such as vertebral fractures and infection. The authors demonstrated that both acquired and synthetic STIR sequences were diagnostically equivalent; five radiologists (four subspecialists and one general radiologist) had similar interobserver agreements for both the conventional and AI-generated sequences for the detection of three findings (prevertebral fluid collections, fracture-related bone edema, and posterior soft tissue/ligamentous injury) against the reference standard. Additionally, synthetic images had a higher mean image quality score. Nonetheless, the authors acknowledged that artifacts in the input images could potentially affect synthetic image quality [42]. Thus, while synthetic MRI sequences could also help reduce scan times, further work is necessary to better understand the effects of MRI artifacts, such as metal or susceptibility artifacts on such algorithms.

Furthermore, certain DL models such as generative adversarial networks (GANs) have been successfully deployed to generate MRI-like images from CT data, and vice versa [41,59]. Gotoh et al. (2022) utilized a conditional GAN (pix2pix) to generate synthetic T2-weighted MRI images from lumbar spine CTs. They achieved a modest peak signal-to-noise ratio of 18.4 dB, although, on qualitative evaluation by two radiologists, there was no significant difference in the image quality with conventional MRI images [59]. These models can be potentially useful for patients who have contraindications to MRI, such as non-MRI compatible implants.

### 3.3. Segmentation

Segmentation comprised the second highest proportion of studies reviewed. Notably, many early studies concentrated on segmentation. Various regions of the spine, including the vertebrae, intervertebral discs, and spinal cord, have been studied for this application. Recently, more sophisticated and complex models have been employed to achieve higher levels of accuracy across a broader range of tasks. Mohanty et al. (2023) demonstrated the use of a novel segmentation technique that initially segments the spinal cord into different regions. A combination of multiple mask regional CNNs (MRCNNs) is then used for each spinal cord segment, which provides a higher overall accuracy of 99% compared to accuracies of 81–96% for the other models (a CNN, deep neural network, and statistical parametric mapping) [45]. Newer models are also able to segment a larger number of structures, improving granularity. Yilizati-Yilihamu et al. (2023) employed a SAFNet-based model to segment 17 unique spinal structures, overcoming issues posed by intra- and inter-class differences across a range of spinal levels. This method extracted low-, mid- and high-level features on MRI images which were then processed separately before being concatenated. The model achieved an overall mean Dice score of 80% against a radiologist, surpassing other models whose Dice scores ranged from 75–79%, with 3D UNet performing the worst. However, SAFNet exhibited relatively poor segmentation for certain structures, such as the L5 vertebra and sacrum. While SAFNet had high Dice scores on most vertebrae, it struggled with accurately depicting the borders of L5 and the sacral intervertebral discs [36]. The sacrum’s unique shape and poorly formed intervertebral discs may have contributed to these difficulties. In contrast, models like 3D DeepLabv3 and ResUNet, which employ superior boundary detection techniques, were more successful in achieving accurate segmentation in these challenging areas. Despite these challenges, SAFNet demonstrated better generalization and overall performance, making it a robust choice for segmentation tasks.

Interpreting MRIs with spinal abnormalities presents a significant challenge for accurate segmentation due to distorted anatomy and altered relationships between normal structures. To address this, several models have been developed for segmentation in specific clinical scenarios, such as spinal cord trauma. Specifically, Masse-Gignac et al. (2023) employed an attention-gated U-net to segment injured spinal cords. The attention gating mechanism helped the architecture focus on the most relevant features while reducing the number of feature maps, leading to a considerable Dice score of 0.95, even in the presence of distorted segmentation boundaries [35].

Additionally, segmentation has been expanded beyond normal anatomical structures to include pathology itself. For instance, Lemay et al. (2021) trained a cascaded neural network for segmentation of intramedullary tumors across different spinal regions. The study included 343 patients with various tumors (namely, astrocytomas, ependymomas and hemangioblastomas) and utilized T2-weighted and T1-weighted post-contrast images. A Dice score of 62% was achieved for segmentation of the tumor itself compared to radiologists’ segmentation, with a higher Dice score of 77% when the tumor cavity and edema were also included. Compared to a single model architecture (a 3D U-net), the cascaded architecture demonstrated increased Dice scores of 30% for edema, and 5% for tumor and tumor cavity [61]. The segmentation of pathology is potentially useful in clinical practice to allow more accurate quantification and post-treatment follow-up.

### 3.4. Diagnostics

There has been considerable interest in using AI for diagnostic applications in spine MRI, and this represented the largest proportion of studies in our review. To provide more focus, we have further categorized the studies based on the type of disease examined.

#### 3.4.1. Degenerative Disease

Degenerative disease along the spine is found in a sizeable proportion of all MRIs performed. Multiple studies have investigated the use of AI models in the detection and classification of degenerative pathologies. These mainly focused on the cervical [20,38,63] and lumbar [33,47,64,68] spine, given the relatively higher incidence in these regions compared to the semi-rigid thoracic spine [1]. Models have also been utilized to allow for the detection of specific pathologies on MRI, such as OPLL [25], which is typically assessed on CT.

A number of studies have focused on identifying the sites of spinal cord or nerve compression. Merali et al. (2021) trained a CNN (ResNet50) to classify cervical spine MRIs for the presence or absence of cord compression on axial T2-weighted images, achieving a high AUC of 0.94. While this model could be used to quickly classify patients with high accuracy, the authors noted that a more precise model that stratifies the severity of spinal cord compression (for example, partial versus circumferential compression, with the latter being more severe) would offer greater clinical utility [63].

In a study by Hallinan et al. (2021), the authors examined the ability of a CNN-based model to perform automated grading of lumbar spinal stenosis at different regions of interest. The model was trained on axial T2-weighted and sagittal T1-weighted sequences and achieved high levels of agreement compared with the reference standard (an expert radiologist with 31 years of experience). Its performance was comparable to that of subspecialist radiologists for dichotomous grading at the central canal (κ = 0.96 versus 0.98 for radiologists) and lateral recesses (κ = 0.92 versus 0.92–0.95 for radiologists) but slightly lower at the neural foramina (κ = 0.89 versus 0.94–0.95 for radiologists) [64]. In a follow-up study, Lim et al. (2022) assessed whether this model could enhance radiologist performance. The authors evaluated the performance of eight radiologists (comprising subspecialists, general radiologists, and in-training radiologists) with and without DL model assistance. They found that DL model assistance generated significant time savings (reduced interpretation time by 76–203 s, *p* < 0.001), with the greatest benefit for in-training radiologists. DL-assisted readers had improved or similar performance compared to the baseline [53]. Such studies that assess the real-world impact of DL models are useful in identifying the areas of greatest benefit and potential problems. In this context, using AI alongside radiologists during image interpretation has the potential to enhance both the efficiency and consistency of reporting by reducing variability in their assessments.

Other studies have focused on specific degenerative pathologies, such as intervertebral disc degeneration. Liawrungrueang et al. (2023) trained a CNN (YOLOv5) to classify lumbar discs on sagittal T2-weighted images using the Pfirrmann classification system [69], a widely used system for communicating the severity of disc degeneration and destruction. Compared to a musculoskeletal radiologist, the model achieved accuracies of more than 95% [47]. Recent work by Xie et al. (2024) employed a combined model using MedSAM followed by radiomics analysis to perform Pfirrmann grading for degenerate cervical discs. The model was trained on sagittal T1- and T2-weighted images and achieved an AUC of 0.95 on a test set, compared against an orthopedic radiologist. It demonstrated the highest accuracy of 90% when using T1- and T2-weighted images in combination (versus 81–86% when trained on either sequence alone) [20]. The ability to classify degenerative pathologies using standardized grading systems can allow for the rapid identification of cases with more severe disease. The use of established criteria would also help facilitate communication among different specialists.

#### 3.4.2. Neoplastic Diseases

Neoplastic disease can affect different structures along the spine including the vertebral column and epidural space with risk of spinal cord compression, potentially leading to significant disability. Bone neoplasms include metastases, myeloma, and primary neoplastic lesions [70]. Additionally, neoplasms can involve the thecal sac/dura and spinal cord. Several models have been utilized to address diagnostic challenges in spine oncology [32,49,51,65], facilitating the distinction between different pathologies with overlapping imaging characteristics. For example, Zhuo et al. (2022) employed the MultiResUNet and DenseNet121-based models to differentiate demyelinating disease from neoplasms (namely ependymoma and astrocytoma) on sagittal T2-weighted MRI alone, without contrast-enhanced sequences. Scans were evaluated by seven neuroradiologists, with the model achieving high accuracies of 79–96% (AUC 0.85–0.99), which was similar or superior to the performance of the neuroradiologists (accuracies of 67–97%). Accuracy for differentiating between the types of demyelinating diseases (multiple sclerosis versus neuromyelitis optica spectrum disorders) was the lowest. This pipeline could potentially be useful for cases where intravenous gadolinium contrast is contraindicated, for example, in patients with renal impairment. However, the authors also performed lesion segmentation and noted relatively poor Dice scores of 0.50–0.58 for the segmentation of demyelinating lesions (versus Dice scores of 0.77–0.80 for neoplasms), suggesting that further work in this area is necessary before AI-based quantification can be applied to clinical practice [49].

Other models have been applied to evaluate complications resulting from neoplastic diseases. For instance, Liu et al. (2023) used a Two-Stream Compare and Contrast Network (TSCCN) model to differentiate between benign and malignant vertebral compression fractures on sagittal T1-weighted and T2-weighted fat-saturated images, a common diagnostic dilemma. In clinical practice, malignant fractures (usually due to metastases) may require surgical management, and the primary malignancy must also be sought. In their study, all cases of malignant fractures were confirmed histologically (total of 14 cancer types). The model achieved higher accuracies of 90–96% (highest accuracy using both MRI sequences in combination) relative to clinical radiologists (accuracies of 81–90%). The TSCCN model does not require the manual segmentation of fractures and allows for the rapid identification of malignant fractures. The authors, however, acknowledge that the generalizability of the model may be limited for other cancer types not included in the study [46].

Additionally, in routine practice, radiologists also evaluate the extent of spinal cord compression resulting from neoplastic disease as it helps guide management and identify patients at risk of neurologic compromise. To this end, Hallinan et al. (2022) used a CNN to grade the severity of metastatic epidural spinal cord compression (MESCC) using the Bilsky classification on axial T2-weighted images. Compared against an experienced musculoskeletal radiologist as the reference standard, the model achieved an almost-perfect agreement for dichotomous grading on internal validation (κ = 0.92) and external testing (κ = 0.94). It was also compared to three clinicians who had similar performance (κ-range = 0.94–0.98). This could be used to identify patients with severe cord compression for prompt specialist review [54]. In a separate study, the authors also demonstrated the feasibility of automated MESCC grading (normal/low/high-grade) on a matched set of contrast-enhanced CT images that had corresponding MRIs. The CT model had a high agreement (κ = 0.87–91) with the expert and was superior to two radiologists (κ = 0.73–0.82). This would potentially allow for even earlier diagnosis on staging CT scans which are routinely performed for patients with cancer [71,72,73].

#### 3.4.3. Infection

MRI is the modality of choice for evaluating spondylodiscitis, allowing for an accurate diagnosis, characterization of the extent of infection, and assessment of complications. However, infections can present a diagnostic challenge as degenerative or inflammatory lesions may exhibit similar MRI findings.

Mukaihata et al. (2023) developed an algorithm to differentiate pyogenic spondylitis from Modic endplate changes, a common diagnostic dilemma. Using a CNN backbone, the authors assessed the model’s performance on sagittal T1-, T2-weighted, and STIR images against a radiologist and specialist orthopedic and spine surgeons. The model demonstrated comparable performance to the clinicians and had a high AUC (0.94–0.95) [48]. Additionally, MRI can be useful in suggesting the likely causative organism for spine infections, helping guide treatment and follow-up. Several studies have applied AI to this effect [37,69]. Wang et al. (2023) evaluated a combined model to predict whether Brucella or Tuberculous spondylitis was more likely using sagittal T1-, T2-, T2-weighted fat-saturated, and axial T2-weighted sequences. Various AI models were used to assess images against the reference standard (defined by clinical and microbiological diagnostic criteria). A random forest model achieved the highest AUC of 0.95, higher than a support vector machine (AUC 0.90–0.94) [37]. Such models are potentially useful as the choice of microbiological therapy and management strategy differs significantly between these conditions.

#### 3.4.4. Trauma

MRI is often used in the assessment of traumatic injuries, providing a detailed visualization of soft tissues including the spinal cord and vertebrae, aiding in the detection of subtle injuries and fractures crucial for accurate diagnosis and treatment planning. Wang et al. (2024) demonstrated that CNNs (YoloV7 and ResNet50) may be used to evaluate for acute vertebral fractures. In their study, sagittal STIR images were used, with the model demonstrating a high accuracy of 98% (sensitivity of 98%, specificity of 97%) against assessments by spine surgeons. While the performance on an external dataset was slightly poorer, this was still relatively high at 92% (sensitivity of 93%, specificity of 92%) [21].

In another application, Jo et al. (2023) developed a two-step algorithm (Attention U-net and Inception-ResNet-V2) for the diagnosis and localization of posterior ligamentous complex injury in patients with thoracolumbar fractures on midsagittal T2-weighted fat-saturated images, which can be particularly difficult for inexperienced readers. Assessment by two experienced musculoskeletal radiologists was used as the reference standard. The algorithm demonstrated comparable performance (AUC 0.93 on internal testing, 0.92 on external testing) to a musculoskeletal radiologist (AUC 0.93), and higher performance than a radiology trainee (AUC 0.83). In addition, they showed that the performance of the radiology trainee was significantly improved when aided by the model (improved from AUC 0.83 to AUC 0.92) [28]. Such models can be used to improve diagnostic confidence for junior readers.

#### 3.4.5. Spondyloarthropathy

MRIs play an important role in the diagnosis and monitoring of spondyloarthropathies, such as ankylosing spondylitis, given its increased sensitivity over conventional techniques like radiographs and CT. It can identify lesions in the pre-clinical stage of the disease and guide the decision on the use of disease-modifying drugs. Tas et al. (2023) demonstrated the use of a multi-stage CNN-based model (termed “ASNet”) in the diagnosis of ankylosing spondylitis with high accuracy (96–100%) on both non-contrast (axial and coronal STIR) and contrast-enhanced T1-weighted MRI sequences. All the included ankylosing spondylitis patients had a clinico-radiological diagnosis and were on follow-up with a rheumatologist. The authors achieved higher accuracies compared to previous similar studies which used ResNet and U-net models (accuracies of 88–92%). Of note, the authors demonstrated the highest accuracy with non-contrast images (99% on coronal and 100% on axial images), which may obviate the need for intravenous contrast in the future [34]. This could improve diagnosis for patients with contraindications such as contrast medium allergy or impaired renal function. In another sample of 330 patients with axial spondyloarthritis, Lin et al. (2024) employed a UNet-based model to detect inflammatory lesions on sagittal STIR images, using combined assessment by an experienced radiologist and rheumatologist as the ground truth. The DL model demonstrated similar results (sensitivity 80%, specificity 88%, on a per-image basis) to a radiologist of four years’ experience (sensitivity 82%, specificity 87%) [23].

### 3.5. Treatment Planning, Patient Selection, and Prognostication

Another growing application of AI is its use in patients who are being evaluated for specific treatments. Models have been developed to predict outcomes for patients undergoing various spinal procedures or surgeries, such as lumbar disc surgery [31], lumbar nucleoplasty [44], and cervical spine surgery [56,60]. Of note, Goedmakers et al. (2021) employed three CNNs (VGGNet19, ResNet19, and ResNet50) to predict which patients would develop adjacent segment disease (on clinical and radiologic follow-up) after undergoing cervical radiculopathy surgery (anterior discectomy and fusion). Conventionally, the prediction of this relatively common complication relies on subjective clinical assessment. The authors used sagittal T2-weighted images and demonstrated a higher accuracy of 95% (using ResNet50) compared to 58% by the clinicians (a neurosurgeon and neuroradiologist). This model offers to provide useful prognostic information and can guide decisions on patient selection, although future work could also account for other variables such as patient demographics and surgical technique [60].

Apart from surgery, other treatments can also be analyzed using predictive algorithms. Chen et al. (2023) investigated an ML-based radiomics algorithm to evaluate sagittal T1-, T2-weighted, STIR, and axial T2-weighted MRIs for radiotherapy prognostication. Follow-up data on tumor progression were classified into “progressive disease” and “non-progressive disease” groups based on established tumor response criteria. The clinical model achieved an AUC of 0.73 (based on features such as multiplicity of tumors, Bilsky score, symptoms) whereas a combined clinical–radiomics model had an improved AUC of 0.83. Although this study was relatively small, with only 52 lesions in the progressive disease group, and benefits over the conventional model were relatively modest, it shows the potential applicability of radiomics models to assist radiation oncologists in treatment selection for difficult cases [30].

### 3.6. Others

A variety of other applications exist. These include various non-interpretative tasks such as vetting MRI requests. Alanazi et al. (2022) compared the performance of experienced radiographers to various AI models in determining whether a lumbar spine MRI request was indicated or not. A random forest model was found to achieve the highest area under the curve of 0.99 [52]. Further models have also been applied to tasks such as processing radiology reports. For instance, Jujjavarapu et al. applied various natural language processing methods to analyze lumbar spine radiograph and MRI reports. High accuracy was achieved (AUC 0.96 with n-grams) for the identification of 26 radiologic findings. The authors also showed reliable extraction of potentially clinically important findings (AUC 0.95 with document embeddings). Such models could be employed to facilitate early clinical review for patients with time-critical pathology [58]. 

### 3.7. AI and ML Techniques

The reviewed studies employed various AI and ML techniques, with CNNs being the most frequently used, both appearing in studies from the earlier years to the most recent. CNNs are versatile and have been applied across a wide range of tasks, including segmentation, detection, and classification. In more recent years, advanced CNN architectures (such as ResNet, DenseNet, YOLO) have been developed. These architectures incorporate new mechanisms to overcome several limitations of conventional models (including the vanishing gradient problem and overfitting), allowing for deeper networks and better performance [21,25,27,28,33,34,45].

U-nets are another common technique, being particularly favored in image segmentation tasks for their precision and efficiency. In later studies, U-net variants (such as 3D U-net, Attention U-net, and MultiRes U-net) were employed to further enhance their capabilities [19,28,49,57]. For instance, 3D U-nets enable the segmentation of volumetric data like MRI scans, preserving spatial information across slices and leading to greater accuracy.

Some studies also implemented ensemble models, where outputs from multiple individual models are aggregated to produce superior results than each model alone. Common ensemble models in this review include Random Forest and boosting models (XGBoost, AdaBoost), which were used to improve the performance for advanced classification tasks such as predicting outcomes after spinal surgery or stereotactic radiotherapy [30,31,39,44].

Overall, there was a noticeable transition from simple, single-model approaches to more sophisticated models over time. Hybrid and ensemble models became increasingly common, reflecting the need for more robust models capable of effectively handling complex tasks.

## 4. Discussion

AI and ML in spine MRI have the potential to address several shortcomings of conventional technology and assessment. However, there remain important gaps and limitations that need to be addressed and studied.

As previously alluded to, one of the major challenges that modern radiology departments face is the significant time required to perform MRIs, despite ongoing advances in MRI technology, acceleration techniques, and pulse sequences. The ability of DL reconstructions to significantly reduce imaging time has the potential to improve scanner utilization and patient comfort [74]. These are pertinent issues, given the increasing demand for advanced imaging such as MRI and the longer examination times compared to other imaging modalities. Many DL reconstruction algorithms promise minimal to no degradation of image quality, ensuring high diagnostic accuracy, and some are already commercially available. In particular, the synthetic MRI sequences generated from CT images could allow clinicians to leverage the speed of the CT with the superior soft tissue resolution of MRI, facilitating more accurate diagnoses for patients who cannot undergo MRI (e.g., patients with MRI-incompatible implants or claustrophobia) [59]. Conversely, synthetic MRI-generated CT sequences can eliminate radiation exposure. However, there are limitations to the current DL technology. Reconstruction algorithms, especially those used for denoising, can exaggerate artifacts and may cause instability in output images, potentially leading to small lesions being overlooked [74,75].

Advances in spine segmentation have served as an important foundation on which more complex applications can be built. Most recently, models developed to segment pathology have given rise to new clinical applications [61]. The segmentation of other diseases such as neoplasms could also lead to more objective and accurate monitoring for treatment response. Further work in this area is necessary to determine the impact on patient outcomes.

With advances in diagnostics, interpretative tasks conventionally performed by trained radiologists can be augmented by AI. An important area of potential impact is increased efficiency and productivity leading to time savings. Given the ever-increasing radiology workloads, AI could potentially be used to reduce interpretation times and reader fatigue, allowing radiologists to focus on more complex cases and patient care [76]. Additionally, AI tools may supplement radiologists by improving their diagnostic accuracy. AI augmentation has been shown to improve radiologists’ performance, particularly those with less experience [28,53]. Another area of particular interest is the synergy between radiomics and deep learning. This field involves quantitative image analysis, offering more precise and accurate lesion characterization or classification than what is possible by human readers alone [20,77]. Additionally, AI models, such as those applied to spondyloarthropathy [34], have the potential to reduce the need for intravenous gadolinium contrast, which is commonly used to enhance diagnostic quality in MRI scans. This reduction could lead to significant cost savings and minimize the potential risks of gadolinium toxicity.

In the field of treatment planning, patient selection, and prognostication, predictive algorithms have shown promise in allowing for the better anticipation of patient outcomes and complications. Spinal surgery and interventions carry significant risks and should be offered to patients who are likely to benefit the most. While existing clinical decision support and predictive tests are available, these often lack consensus and can have conflicting evidence [78,79]. AI systems that accurately predict outcomes can improve patient care and resource optimization.

However, despite widespread optimism about the purported benefits of these AI technologies, there are important limitations and potential areas for further study, which we will address in the next sections.

### 4.1. Generalizability

Generalizability refers to the ability of an AI model to perform its intended function on a new set of data that was not part of the model development process [80]. While models may exhibit high levels of accuracy on test sets, developing a generalizable model presents unique challenges. Ethical, legislative, and practical concerns often lead to the development of models based on patient data from a single healthcare institution or country. Variability in institutional imaging protocols, MRI equipment, and pulse sequences introduces significant challenges to consistent AI model performance. In addition, variations in treatment approaches further complicate the development of generalizable models. These factors collectively limit the performance of AI models when applied in different settings [81,82].

Several strategies can be employed to improve generalizability. Firstly, ensuring the availability of data from varied populations is crucial. For instance, Xu et al. (2023) used a large training dataset to develop an AI model for thyroid nodule classification on ultrasound. They utilized data from 10,023 patients across 208 hospitals and 12 equipment vendors, achieving a high AUC of 0.90. The use of scans from a heterogeneous patient population was cited as an important factor for the model’s strong performance [83]. Large medical image datasets, including RadImageNet, MedPix, CheXpert, have been made available in recent years. Additionally, the Radiological Society of North America (RSNA) and the American Society of Neuroradiology (ASNR) recently launched a publicly available dataset of cases annotated by 50 expert radiologists across eight institutions, with the goal of encouraging the development of AI tools for degenerative lumbar spine MRIs [84]. Improving the availability of diverse, high-quality data can potentially overcome some of the challenges posed by limited diversity in training datasets, potentially resulting in more robust models.

Secondly, techniques such as transfer learning can be employed. Transfer learning, which includes domain adaptation, involves making modifications to a model in order to improve its performance on previously unseen tasks [85]. Xuan et al. (2023) employed transfer learning on CNNs (YOLOv3, YOLOv5, PP-YOLOv2) that were pre-trained on general image datasets. Transfer learning was applied by using these models to train a CNN, together with sagittal T2-weighted MRI images labeled by experienced spinal surgeons for evaluation of various features (including disc bulges and spondylolisthesis). The model had a higher accuracy (98%) compared to three spine doctors (accuracies ranging from 70 to 88%) [86]. Similar approaches could be applied to other spine AI models, ensuring their validity when applied to varied settings.

Thirdly, “stress testing” involves evaluating an AI model under varied or extreme conditions to identify potential weak points [81]. In radiology, this may include modifying the input image by rotating, cropping, or adjusting the brightness. Such tests help simulate clinical variability. Santomartino et al. (2024) recently evaluated a bone age prediction algorithm on external images before and after applying transformations to simulate real-world variations (for example, rotating or flipping the image, altering brightness and contrast). The algorithm performed well on the external dataset with a mean absolute difference of 6.9 months and 16.2% clinically significant errors (CSEs) when compared to radiologists. However, its performance significantly worsened when tested on the altered images; when the image resolution was altered, the mean absolute difference increased to 118.3 months. This process helped demonstrate the important pitfalls of the model [87]. Stress testing allows for the simulation of real-world variations in image quality that can significantly impact model performance.

As more AI models become commercially available, it is crucial that they are rigorously validated before they can be applied to new healthcare settings, ensuring their accuracy and safety for patient use.

### 4.2. Implementation

Implementing AI in clinical practice involves overcoming several hurdles. Most recently, a multi-society statement was released by several radiological organizations (ACR, CAR, ESR, RANZCR, RSNA) that provided guidance on the application of AI tools, from development to implementation and use [88]. The statement highlighted the need for rigorous evaluation to ensure patient safety and supported the integration of AI into existing healthcare information technology (IT) infrastructure. Other authors have emphasized the importance of using vendor-neutral platforms, which can streamline algorithms from multiple developers and facilitate end-user access to AI-generated results [89]. Addressing these concerns will ensure that AI algorithms are reliable and effective.

A key challenge in healthcare is maintaining patient confidentiality while leveraging large volumes of medical data for AI training and deployment. Privacy concerns arise due to the sensitive nature of medical data, and the risk of data breaches or misuse could compromise patient trust and legal compliance. Designing AI systems that protect patient identities through techniques like data anonymization and encryption is essential [90].

The use of AI in healthcare settings such as radiology also raises ethical concerns. Patient well-being, equity, privacy, and dignity should be prioritized. Responsible use of AI includes ensuring that patient data are properly regulated and kept secure. AI models may also exaggerate pre-existing biases in healthcare, particularly the effects of selection bias. Such bias is inadvertently introduced when algorithms are applied to patient data that differ from the data on which they were trained, where the incidence of various pathologies may differ. For example, an AI model trained primarily on data from urban hospitals may underperform when used in rural settings, where the prevalence of certain conditions and patient demographics differ significantly. Even within a single health system, AI systems may inadvertently introduce bias against minority populations due to unrepresentative training data. This can potentially lead to less accurate predictions for certain groups and increase healthcare disparity. Minimizing bias is crucial to ensuring fairness and accuracy [88,90,91,92,93,94].

Trust is another key factor in the successful implementation of AI models into clinical care. Radiologists’ trust in AI has been identified as one of the most common barriers to its adoption [95]. Many commonly used algorithms operate as “black box” solutions, making it difficult to fully understand how conclusions are derived. Building patient trust in AI is also important for its role to be widely accepted in patient care. Clear communication about how AI works and its benefits can foster this trust. Involving end-users and patients in the AI development process and addressing their concerns is also vital [96]. For example, saliency maps (heat maps) can be used to improve model explainability. These are visual representations that highlight parts of the image that are most relevant to a DL model’s predictions. Brima and Atemkeng (2024) used various saliency methods (GradCAM, ScoreCAM, XRAI) on datasets comprising MRI scans depicting brain tumors and COVID-19 chest radiographs. Employing both qualitative visual assessment and quantitative (Accuracy and Softmax Information Curves) metrics, they showed that saliency maps can offer accurate representations of a model’s decision-making process [97].

Ongoing research and close collaboration between researchers, healthcare providers, and regulatory bodies is necessary to ensure the smooth implementation of AI in routine practice. Establishing robust regulatory frameworks and continuously monitoring AI systems will be essential to addressing any emerging issues and maintaining high standards of patient care.

### 4.3. Study Limitations

While this review provides a comprehensive analysis of the current applications of AI and ML in spine MRI, several limitations should be acknowledged. Firstly, the heterogeneity of the included studies and lack of inferential statistics limit the robustness and ability to draw generalized conclusions. Differences in study designs, patient populations, MRI protocols, and models make it difficult to directly compare outcomes between studies. Nonetheless, we sought to provide a comprehensive overview across the range of applications that have been studied in this field, with the aim of highlighting current trends and identifying key gaps in the literature which could serve as targets for further research. Secondly, a majority of the studies reviewed were retrospective in nature, which limits the ability to assess the real-world applicability of these AI tools in prospective clinical settings. Another notable limitation is the lack of standardized evaluation metrics across studies, making it challenging to objectively compare the performance of different AI models. Finally, the review did not consider the potential biases that could be introduced by AI models, such as those related to patient demographics, which could impact the fairness and equity of AI-driven healthcare solutions.

### 4.4. Proposed Areas of Future Research

Firstly, the exploration of foundation AI models in healthcare presents an exciting opportunity. These models are trained on varied and much more extensive datasets than the conventional models, making them adaptable for a wide range of tasks. Furthermore, they have the potential to integrate multiple data types to provide comprehensive insights. However, there are several limitations, including the increased complexity in training and validating these generalist models [98,99]. Thus, further work is necessary to evaluate potential applications in spine MRI.

One such application is the development of comprehensive models for image interpretation. Such models have already been developed and validated for applications like interpreting chest radiographs, where the detection of multiple pathologies is possible [100,101,102]. However, in this application, many existing models focus on detecting a single pathology or differentiating between a small number of pathologies. Currently, many of these models still require radiologist input for image interpretation. Comprehensive AI models that can detect and classify a wide range of pathologies—including degenerative disease, fractures and vertebral malalignment, marrow signal abnormality, spinal cord abnormalities and incidental extra-spinal findings—will significantly enhance the diagnostic process. These advanced models could also integrate information from the patient’s electronic medical records. Multidisciplinary collaboration among computer scientists, radiologists, and clinicians is necessary to identify areas where clinically relevant models can provide the greatest benefit for patients [103].

Another area where foundation models can improve patient care is through large language models (LLMs). Privacy-protecting LLMs (PP-LLMs), which can manage large volumes of healthcare data while ensuring patient privacy and security, are particularly promising. In radiology, LLMs have already been employed for a range of tasks. For example, they have been used to determine the most appropriate imaging protocol for different types of scans [104], generate radiology reports, and summarize reports [105,106]. Spine MRI reports are a suitable area for LLM assistance as they are typically structured by spinal level, which can make the reporting process tedious and time-consuming. LLMs could automate much of this work, generating consistent and accurate reports more efficiently than manual methods. In addition, LLMs have the potential to enhance the understanding of radiological findings by both referring clinicians and patients. LLMs can help translate complicated medical jargon into language that is accessible to patients, helping them to better understand their diagnosis [107,108]. As the performance of LLMs continues to improve, their range of applications in healthcare is likely to expand. Future advancements could include more varied diagnostic tasks. In particular, recent multimodal LLMs which can interpret both text and images are particularly useful in radiology, with the potential to aid in tasks such as identifying errors in radiology reports [109]. Such algorithms could be developed for spine imaging thus ensuring patient safety.

A final key area of future research is the evaluation of real-world applications of AI, particularly its impact on diagnostic accuracy, productivity, and patient outcomes. Studies like those by Lim et al. (2022), where the radiologist’s performance in interpreting the lumbar spine MRI was assessed with and without a DL model, provide valuable insight. The largest improvements in time and accuracy were seen for in-training and general radiologists, with subspeciality radiologists achieving the least productivity gains [53]. Studies on the application of AI systems for other diseases have helped demonstrate unexpected or unintended consequences. A recent study by Yu et al. (2024) examined the impact of AI assistance on 140 radiologists who were tasked with interpreting chest radiographs. The authors found that the impact of AI assistance on radiologist performance was variable, and that AI errors significantly impacted treatment outcomes [110]. Similar studies will be useful in evaluating other interpretative tasks, such as spine imaging, to understand the influence of AI more holistically.

## 5. Conclusions

Our review has highlighted the significant potential that AI and ML have in revolutionizing spine MRI by addressing important challenges in image acquisition, diagnostic accuracy, and treatment planning. We have examined key advancements in AI including the development of DL reconstruction algorithms that allow for faster image acquisition, and models that allow for improved diagnostic performance. We also acknowledge the limitations of current AI technology. Future work will require collaborative efforts to fully exploit new technologies and address practical challenges related to generalizability and implementation.

## Figures and Tables

**Table 1:** Summary of Selected Studies.

No	Study Title	Authorship	Year of Publication	Journal Name	Application and Primary Outcome Measure	Sample Size *	Spine Region Studied	MRI Sequences Used	Artificial Intelligence Technique Used	Key Results and Conclusion
1	A quantitative evaluation of the deep learning model of segmentation and measurement of cervical spine MRI in healthy adults	Y Zhu et al. [19]	2024	J Appl Clin Med Phys	Segmentation of cervical spine structures (subarachnoid space area/diameter, spinal cord area/diameter, anterior and posterior extra-spinal space)	160	Cervical	Sagittal T1w, T2w, axial T2w	3D U-net	No comparative statistics
2	MRI radiomics-based decision support tool for a personalized classification of cervical disc degeneration: a two-center study	J Xie et al. [20]	2024	Front Physiol	Classification of cervical disc degeneration (Pfirrmann grading)	435	Cervical	Sagittal T1w, T2w	MedSAM	Disc segmentation Dice 0.93. Random forest overall performance AUC 0.95, accuracy 90%, precision 87%
3	A deep-learning model for diagnosing fresh vertebral fractures on magnetic resonance images	Y Wang et al. [21]	2024	World Neurosurg	Detection of fresh vertebral fractures	716	Whole spine	Midsagittal STIR	YoloV7, Resnet 50	Accuracy 98%, sensitivity 98%, specificity 97%. External dataset accuracy 92%
4	Diagnostic evaluation of deep learning accelerated lumbar spine MRI	KM Awan et al. [22]	2024	Neuroradiol J	Comparison of deep learning accelerated protocol to conventional protocol for neural stenosis and facet arthropathy	36	Lumbar	Sagittal T1w, T2w, STIR, axial T2w	CNN	Non-inferior in all aspects however reduced signal-to-noise ratio and increased artifact perception. Interobserver variability κ = 0.50–0.76
5	A deep neural network for MRI spinal inflammation in axial spondyloarthritis	Y Lin et al. [23]	2024	Eur Spine J	Detection of inflammatory lesions on STIR sequence for patients with axial spondyloarthritis	330	Whole spine	Sagittal STIR	U-net	AUC 0.87, sensitivity 80%, specificity 88%, comparable to a radiologist. True positive lesion Dice 0.55.
6	Semi-automatic assessment of facet tropism from lumbar spine MRI using deep learning: a Northern Finland birth cohort study	N Kowlagi et al. [24]	2023	Spine (Phila Pa 1976)	Measurement of facet joint angles	490	Lumbar (L3/4 to L5/S1)	Axial T2w	U-net	Dice 0.93, IOU 0.87
7	A convolutional neural network for automated detection of cervical ossification of the posterior longitudinal ligament using magnetic resonance imaging	Z Qu et al. [25]	2023	Clin Spine Surg	Detection of ossification of posterior longitudinal ligament	684	Cervical	Sagittal MRI	ResNet	Accuracy 93–98%, AUC 0.91–0.97. ResNet50 and ResNet101 had higher accuracy and specificity than all human readers
8	Deep learning-based k-space-to-image reconstruction and super resolution for diffusion-weighted imaging in whole-spine MRI	DK Kim et al. [26]	2024	Magn Reson Imaging	K-space-to-image reconstruction for whole spine DWI in patients with hematologic and oncologic diseases	67	Whole spine	Axial single-shot echo-planar DWI	CNN	Higher diagnostic confidence scores and overall image quality
9	Automatic detection and classification of Modic changes in MRI images using deep learning: intelligent assisted diagnosis system	Gang L et al. [27]	2024	Orthop Surg	Detection and classification of Modic endplate changes	168	Lumbar	Median sagittal T1w and T2w	Single shot multibox detector, ResNet18	Internal dataset: accuracy 86%, recall 88%, precision 85%, F1-score 86%, interobserver κ = 0.79 (95%CI 0.66–0.85). External dataset: accuracy 75%, recall 77%, precision 78%, F1-score 75%, interobserver κ = 0.68 (95%CI 0.51–0.68)
10	Deep learning system for automated detection of posterior ligamentous complex injury in patients with thoracolumbar fracture on MRI	SW Jo et al. [28]	2023	Sci Rep	Detection of posterior ligamentous complex injury in patients with acute thoracolumbar fractures	500	Thoracic and lumbar	Midline sagittal T2w	Attention U-net and Inception-ResNetv2	AUC 0.92–0.93 (vs. 0.83–0.93 for radiologists)
11	Cross-sectional area and fat infiltration of the lumbar spine muscles in patients with back disorders: a deep learning-based big data analysis	J Vitale et al. [29]	2023	Eur Spine J	Segmentation of lumbar paravertebral muscles and correlation with age	4434	Lumbar	Axial T2w	U-net	Higher cross-sectional area in males (*p* < 0.001). Positive correlation between age and total fat infiltration (r = 0.73, *p* < 0.001), negligible negative correlation between cross-sectional area and age (r = −0.24, *p* < 0.001)
12	MRI feature-based radiomics models to predict treatment outcome after stereotactic body radiotherapy for spinal metastases	Y Chen et al. [30]	2023	Insights Imaging	Prediction of treatment outcome after stereotactic body radiotherapy for spine metastasis	194	Whole spine	Sagittal T1w, T2w, STIR, axial T2w	Multiple (including AdaBoost, XGBoost, RF, SVM)	Combined model AUC 0.83, clinical model AUC 0.73
13	Clinical and radiomics feature-based outcome analysis in lumbar disc herniation surgery	B Saravi et al. [31]	2023	BMC Musculoskelet Disord	Combination of radiomics features and clinical features to predict lumbar disc herniation surgery outcomes	172	Lumbar	Sagittal T2w	Multiple (including XGBoost, Lagrangian SVM, RF radial basis function neural network)	Accuracy 88–93% (vs. 88–91% for clinical features alone)
14	Differentiating spinal pathologies by deep learning approach	O Haim et al. [32]	2024	Spine J	Differentiation of spinal lesions into infection, carcinoma, meningioma and schwannoma	231	Whole spine	Variable (T2w, T1w post-contrast)	Fast.ai	Accuracy 78% (validation), 93% (test)
15	Deep learning-based detection and classification of lumbar disc herniation on magnetic resonance images	W Zhang et al. [33]	2023	JOR Spine	Detection and classification of lumbar disc herniation according to the Michigan State University classification	1115	Lumbar	Axial T2w	Faster R-CNN, ResNeXt101	Internal dataset: detection IOU 0.82, classification accuracy 88%, AUC 0.97, interclass correlation 0.87. External dataset: detection IOU 0.70, classification accuracy 74%, AUC 0.92, interclass correlation 0.79
16	ASNET: a novel AI framework for accurate ankylosing spondylitis diagnosis from MRI	NP Tas et al. [34]	2023	Biomedicines	Prediction of ankylosing spondylitis diagnosis on MRI	2036	Sacroiliac joints	Axial, coronal STIR, coronal T1w post-contrast	DenseNet201, ResNet50, ShuffleNet	Accuracy 100%, recall 100%, precision 100%, F1-score 100%
17	Attention-gated U-Net networks for simultaneous axial/sagittal planes segmentation of injured spinal cords	N Masse-Gignac et al. [35]	2023	J Appl Clin Med Phys	Segmentation of the spinal cord in patients with traumatic injuries	94	All (mainly cervical)	Sagittal T2w	U-Net	Dice 0.95
18	A spine segmentation method based on scene aware fusion network	EE Yilizati-Yilihamu et al. [36]	2023	BMC Neurosci	Segmentation of lumbar spine MRI into individual vertebrae and discs by level	172	Lumbar	Sagittal MRI	Scene-Aware Fusion Network (SAFNet)	Dice 0.79–0.81 (average 0.80)
19	MRI radiomics-based evaluation of Tuberculous and Brucella spondylitis	W Wang et al. [37]	2023	J Int Med Res	Differentiation of Tuberculous spondylitis from Brucella spondylitis, and culture positive from culture negative Tuberculous spondylitis	190	Whole spine	Sagittal T1w, T2w, fat suppressed	RF, SVM	SVM AUC 0.90–0.94, RF AUC 0.95
20	Deep phenotyping the cervical spine: automatic characterization of cervical degenerative phenotypes based on T2-weighted MRI	F Niemeyer et al. [38]	2023	Eur Spine J	Classification of cervical spine into degenerative phenotypes based on disc and osteophyte configuration	873	Cervical	Sagittal MRI	3D CNN	Disc κ = 0.55–0.68, disc displacement κ = 0.58–0.74, disc space narrowing κ = 0.65–0.72, osseous abnormalities κ = 0.18–0.49
21	MRI-based radiomics assessment of the imminent new vertebral fracture after vertebral augmentation	J Cai et al. [39]	2023	Eur Spine J	Evaluation of risk of new vertebral fracture after vertebral augmentation	168	Lumbar	T2w	Multiple (logistic regression, RF, SVM, XGBoost)	AUC 0.90–0.93, superior to clinical features alone (*p* < 0.05)
22	Associations between vertebral localized contrast changes and adjacent annular fissures in patients with low back pain: a radiomics approach	C Waldenberg et al. [40]	2023	J Clin Med	Detection of adjacent level annular fissure based on vertebral changes on MRI	61	Lumbar	Sagittal T1w, T2w, discography, CT	Multilayer perceptron, RF, K-nearest neighbor	Accuracy 83%, sensitivity 97%, specificity 28%, AUC 0.76
23	Imaging evaluation of a proposed 3D generative model for MRI to CT translation in the lumbar spine	M Roberts et al. [41]	2023	Spine J	Generation of 3D CT from sagittal MRI data	420	Lumbar	Sagittal T1w	3D cycle-GAN	Measurements in sagittal plane <10% relative error, axial plane up to 34% relative error
24	Deep learning-generated synthetic MR imaging STIR spine images are superior in image quality and diagnostically equivalent to conventional STIR: a multicenter, multireader trial	LN Tanenbaum et al. [42]	2023	AJNR	Validation of synthetically created STIR images created from T1w and T2w	93	Whole spine	Sagittal T1w, T2w, STIR	CNN	No significant difference between synthetic and acquired STIR, higher image quality for synthetic STIR (*p* < 0.0001)
25	Prediction of osteoporosis using MRI and CT scans with unimodal and multimodal deep-learning models	Y Kucukciloglu et al. [43]	2024	Diagn Interv Radiol	Prediction of osteoporosis on lumbar spine MRI and CT against DEXA scans	120	Lumbar	Sagittal T1w, CT, DEXA	CNN	Accuracy 96–99%
26	Machine learning assisting the prediction of clinical outcomes following nucleoplasty for lumbar degenerative disc disease	PF Chiu et al. [44]	2023	Diagnostics (Basel)	Prediction of pain improvement after lumbar nucleoplasty for degenerative disc disease	181	Lumbar	Axial T2w	Multiple (SVM, light gradient boosting machine, XGBoost, XGBRF, CatBoost, iRF)	Improved RF: accuracy 76%, sensitivity 69%, specificity 83%, F1-score 0.73, AUC 0.77
27	NAMSTCD: A novel augmented model for spinal cord segmentation and tumor classification using deep nets	R Mohanty et al. [45]	2023	Diagnostics (Basel)	Segmentation of spinal cord regions and tumour types	5000 images	Whole spine	Not mentioned	Multiple (Multiple Mask Regional CNN (MRCNNs), VGGNet 19, YoLo V2, ResNet 101, GoogleNet	Classification accuracy 99% (versus 81–96% for other models)
28	Benign vs. malignant vertebral compression fractures with MRI: a comparison between automatic deep learning network and radiologist’s assessment	B Liu et al. [46]	2023	Eur Radiol	Differentiation of benign and malignant vertebral compression fractures	209	Whole spine	Median sagittal T1w, T2w fat suppressed	Two stream compare and contrast network (TSCCN)	AUC 92–99%, accuracy 90–96% (higher than radiologists), specificity 94–99% (higher than radiologists)
29	Automatic detection, classification, and grading of lumbar intervertebral disc degeneration using an artificial neural network model	W Liawrungrueang et al. [47]	2023	Diagnostics (Basel)	Classification of lumbar disc degeneration (Pfirrmann grading)	515	Lumbar	Sagittal T2w	Yolov5	Accuracy > 95%, F1-score 0.98
30	Differentiating magnetic resonance images of pyogenic spondylitis and spinal Modic change using a convolutional neural network	T Mukaihata et al. [48]	2023	Spine (Phila Pa 1976)	Differentiation of Modic changes from pyogenic spondylitis on MRI	100	Whole spine	Sagittal T1w, T2w, STIR	CNN	AUC 0.94–0.95, higher accuracy than clinicians (*p* < 0.05)
31	Automated classification of intramedullary spinal cord tumors and inflammatory demyelinating lesions using deep learning	Z Zhuo et al. [49]	2022	Radiol Artif Intell	Differentiation of cord tumors from demyelinating lesions	647	Whole spine	Sagittal T2w	MultiResU-net, DenseNet121	Test cohort Dice 0.50–0.80, accuracy 79–96%, AUC 0.85–0.99
32	Ultrafast cervical spine MRI protocol using deep learning-based reconstruction: diagnostic equivalence to a conventional protocol	N Kashiwagi et al. [50]	2022	Eur J Radiol	Validation of an ultrafast cervical spine MRI protocol	50	Cervical	Sagittal T1w, T2w, STIR, axial T2*w	CNN	κ = 0.60–0.98, individual equivalence index 95% CI < 5%
33	Differentiation between spinal multiple myeloma and metastases originated from lung using multi-view attention-guided network	K Chen et al. [51]	2022	Front Oncol	Differentiation of multiple myeloma lesions from metastasis on MRI	217	Whole spine	T2w, T1w post-contrast (3 planes)	Multi-view attention guided (MAGN), ResNet50, Class Activation Mapping	Accuracy 79–81%, AUC 0.77–0.78, F1-score 0.67–0.71
34	Development of lumbar spine MRI referrals vetting models using machine learning and deep learning algorithms: Comparison models vs. healthcare professionals	AH Alanazi et al. [52]	2022	Radiography (Lond)	Vetting of MRI lumbar spine referrals for valid indications	1020	Lumbar	Nil	SVM, logistic regression, RF, CNN, bi-directional long-short term memory (Bi-LSTM)	RF AUC 0.99, CNN AUC 0.98 (outperforming radiographers)
35	Improved productivity using deep learning-assisted reporting for lumbar spine MRI	DSW Lim et al. [53]	2022	Radiology	Evaluation of time savings and accuracy for AI-assisted MRI lumbar spine reporting	25	Lumbar	Sagittal T1w, axial T2w	CNN, ResNet101	Reduced interpretation time (*p* < 0.001), improved or equivalent interobserver agreement with DL assistance
36	Deep learning model for classifying metastatic epidural spinal cord compression on MRI	J Hallinan et al. [54]	2022	Front Oncol	Classification of metastatic vertebral and epidural disease (Bilsky classification)	247	Thoracic	Axial T2w	ResNet50	Internal dataset: κ = 0.92–0.98, external dataset: κ = 0.94–0.95
37	Vertebral deformity measurements at MRI, CT, and radiography using deep learning	A Suri et al. [55]	2021	Radiol Artif Intell	Measurement of vertebral deformity on MRI, CT and radiographs	1744	Whole spine	Sagittal T1w, T2w, CT, radiographs	Neural network	Vertebral measurement mean height percentage error 1.5–1.9% ± 0.2–0.4, lumbar lordosis angle mean absolute error 2.3–3.6°
38	Predicting postoperative recovery in cervical spondylotic myelopathy: construction and interpretation of T2*-weighted radiomic-based extra trees models	MZ Zhang et al. [56]	2022	Eur Radiol	Prediction of recovery rate after cervical spondylotic myelopathy surgery based on MRI and clinical features	151	Cervical	T2w, T2*w	Threshold selection, collinearity removal, tree-based feature selection	AUC 0.71–0.81 (vs. conventional clinical and radiologic models AUC 0.40–0.55)
39	Fully automated segmentation of lumbar bone marrow in sagittal, high-resolution T1-weighted magnetic resonance images using 2D U-NET	EJ Hwang et al. [57]	2022	Comput Biol Med	Segmentation of normal and pathological bone marrow on MRI lumbar spine	100	Lumbar	Sagittal T1w	U-net3D, Grow-cut	Healthy subjects Dice 0.91–0.96, diseased subjects Dice 0.83–0.95
40	A comparison of natural language processing methods for the classification of lumbar spine imaging findings related to lower back pain	C Jujjavarapu et al. [58]	2022	Acad Radiol	Classification of spine MRI and radiograph reports into 26 findings	871	Lumbar	MRI, radiographs	Elastic-net logistic regression	AUC 0.96 for all findings (n-grams), AUC 0.95 for potentially clinically important findings
41	Virtual magnetic resonance lumbar spine images generated from computed tomography images using conditional generative adversarial networks	M Gotoh et al. [59]	2022	Radiography (Lond)	Generation of virtual MRI images from CT	22	Lumbar	MRI	Conditional GAN	No significant difference between virtual and conventional MRI, except in visualization of spinal canal structure. Peak signal-to-noise ratio 18.4 dB
42	Deep learning for adjacent segment disease at preoperative MRI for cervical radiculopathy	CMW Goedmakers et al. [60]	2021	Radiology	Prediction of adjacent segment disease after anterior cervical discectomy and fusion surgery on pre-operative MRI	344	Cervical	Sagittal T2w	CNN	Accuracy 95% (vs. 58% for clinicians), sensitivity 80% (vs. 60%), specificity 97% (vs. 58%)
43	Automatic multiclass intramedullary spinal cord tumor segmentation on MRI with deep learning	A Lemay et al. [61]	2021	Neuroimage Clin	Segmentation of three common spinal cord tumors	343	Whole spine	Sagittal T2w, T1w post-contrast	U-net	Dice 0.77 (all abnormal signal), 0.62 (tumour alone), true positive detection > 87% (all abnormal signal)
44	A preliminary study using spinal MRI-based radiomics to predict high-risk cytogenetic abnormalities in multiple myeloma	J Liu et al. [62]	2021	Radiol Med	Prediction of high-risk cytogenic abnormalities in multiple myeloma based on MRI	248 lesions	Whole spine	Sagittal T1w, T2w, T2w fat suppressed	Logistic regression	AUC 0.86–0.87, sensitivity 79%, specificity 79%, PPV 75%, NPV 82%, accuracy 79%
45	A deep learning model for detection of cervical spinal cord compression in MRI scans	Z Merali et al. [63]	2021	Sci Rep	Dichotomous spinal cord compression for cervical spine	289	Cervical	Axial T2w	CNN	AUC 0.94, sensitivity 88%, specificity 89%, F1-score 0.82
46	Deep learning model for automated detection and classification of central canal, lateral recess, and neural foraminal stenosis at lumbar spine MRI	J Hallinan et al. [64]	2021	Radiology	Grading of lumbar spinal canal, lateral recess and neural foraminal stenosis	446	Lumbar	Sagittal T1w, axial T2w	CNN	Recall 85–100%, dichotomous classification κ-range = 0.89–0.96 (vs. 0.92–0.98 for radiologists)
47	A deep convolutional neural network with performance comparable to radiologists for differentiating between spinal schwannoma and meningioma	S Maki et al. [65]	2020	Spine (Phila Pa 1976)	Differentiation of meningioma from schwannoma	84	Whole spine	Sagittal T2w, T1w post-contrast	CNN	AUC 0.87–0.88, sensitivity 78–85% (vs. 95–100% for radiologists), specificity 75–82% (vs. 26–58%), accuracy 80–81% (vs. 69–82%)
48	Quantitative analysis of neural foramina in the lumbar spine: an imaging informatics and machine learning study	B Gaonkar et al. [66]	2019	Radiol Artif Intell	Segmentation and statistical modelling of lumbar neural foraminal area	1156	Lumbar	Sagittal T2w	SVM, U-net	Dice 0.63–0.68 (neural foramen), 0.84–0.91 (disc)
49	Performance of the deep convolutional neural network based magnetic resonance image scoring algorithm for differentiating between Tuberculous and pyogenic spondylitis	K Kim et al. [67]	2018	Sci Rep	Differentiation of pyogenic from Tuberculous spondylitis	161	Whole spine	Axial T2w	Deep CNN	AUC 0.80 (vs. 0.73 for radiologists, *p* = 0.079)
50	SpineNet: automated classification and evidence visualization in spinal MRIs	A Jamaludin et al. [68]	2017	Med Image Anal	Detection and classification of multiple abnormalities (Pfirrmann grading, disc narrowing, endplate defects, marrow changes, spondylolisthesis, central canal stenosis)	2009	Lumbar spine	T2w sagittal	CNN	Pfirmann inter-rater κ = 0.69–0.81, overall accuracy 74%

Artificial intelligence (AI), magnetic resonance imaging (MRI), T1-weighted (T1w), T2-weighted (T2w), T2*-weighted (T2*w), area under the curve (AUC), segment anything model (SAM), convolutional neural network (CNN), short-tau inversion recovery (STIR), intersection over union (IOU), diffusion-weighted imaging (DWI), random forest (RF), support vector machine (SVM), computed tomography (CT), generative adversarial network (GAN), dual-energy X-ray absorptiometry (DEXA). * numbers are patients unless stated otherwise.

## Data Availability

Not applicable.
